# P67 Urinary tract infection treatment outcomes: a systematic review of antibiotic selection and key clinical factors

**DOI:** 10.1093/jacamr/dlag102.073

**Published:** 2026-06-26

**Authors:** Darrin Omer, Rasha Abdelsalam Elshenawy

**Affiliations:** School of Health, Medicine and Life Sciences, University of Hertfordshire, Hatfield AL10 9AB, UK; School of Health, Medicine and Life Sciences, University of Hertfordshire, Hatfield AL10 9AB, UK

## Abstract

**Background:**

Urinary tract infections (UTIs) are among the most common infections in clinical practice, representing a major driver of antibiotic prescribing across primary and secondary care.^1^ Empirical therapy is routinely initiated before microbiological results are available, a practice increasingly complicated by antimicrobial resistance (AMR). Globally, AMR was associated with 4.95 million deaths in 2019, with 1.27 million directly attributable.^2^ In UTIs, resistance reduces first-line agent effectiveness, increasing treatment failure and recurrence risk. Despite this, evidence on the variables influencing empirical antibiotic selection remains fragmented, creating a clear gap this review addresses.^3^

**Objectives:**

To identify clinical and microbiological variables influencing empirical antibiotic selection and UTI outcomes; differentiate uncomplicated from complicated UTI; and assess findings across healthcare settings using the WHO AWaRe classification framework to support evidence-based prescribing.

**Methods:**

This review was conducted in accordance with PRISMA 2020 guidelines. Searches were performed across PubMed, Ovid MEDLINE, SCOPUS, and the Cochrane Library, restricted to English-language studies published between 2016 and 2026 involving adult populations. Studies were eligible if they reported empirical antibiotic prescribing for UTI and at least one clinical treatment outcome. Data were extracted using a structured form covering study characteristics, clinical variables, microbiological findings, and treatment outcomes. All antibiotic regimens were categorized using the WHO AWaRe classification.^5^ No ethical approval was required as no primary data were collected.

**Results:**

Of 847 records identified, 11 studies met the inclusion criteria across diverse study designs, geographic settings, and clinical environments. Access class antibiotics, principally nitrofurantoin and fosfomycin, predominated across the included studies. Nitrofurantoin demonstrated the most favourable efficacy profile, achieving the lowest treatment failure rate of 15.3% and a clinical cure rate of 70% at 28 days in randomized controlled trial evidence. In contrast, fosfomycin showed significantly higher real-world treatment failure rates of 30.1%, highlighting a clinically important discrepancy between trial and practice settings. Watch class antibiotics, including fluoroquinolones and β-lactams, were associated with markedly higher resistance rates, reaching 40.5% for β-lactams, reinforcing concerns regarding their continued empirical use in uncomplicated UTI. The strongest independent predictors of UTI treatment failure were hospital-acquired infection, prior antibiotic use within three months, and MDR pathogens. Additional risk factors included increasing age, recurrent UTI, delirium in elderly patients, obesity, and diabetes mellitus. Inappropriate empirical antibiotic selection was associated with nearly double the treatment failure rate compared with guideline-concordant prescribing.

**Conclusions:**

This systematic review demonstrates that nitrofurantoin is the best-performing first-line Access class antibiotic for uncomplicated UTI, consistently supported by both randomized controlled trial and real-world evidence. Findings reveal a clear advantage of AWaRe-aligned prescribing, with Access class agents yielding superior clinical cure rates and markedly lower resistance profiles compared with Watch class alternatives. Clinical variables, including age, comorbidities, and prior antibiotic use, must be systematically integrated with microbiological factors to optimize empirical antibiotic selection. These findings provide a robust evidence base for structured antimicrobial stewardship strategies to improve UTI outcomes, optimize antibiotic prescribing, combat AMR, and ultimately save patient lives.
